# Supplementary material for: Modeling glioblastoma heterogeneity as a dynamic network of cell states
Source: Mol Syst Biol. 2021 Sep 16;17(9):e10105. doi: 10.15252/msb.202010105 (PMC8444284; doi:10.15252/msb.202010105)
Supplement: Supplementary file 6 — Source Data for Figure 5 [file MSB-17-e10105-s004.zip › Figure5A_sourcedata/GSEA_3071/nosigdb_stateIII.GseaPreranked.1622795155562/NB_KI_CLUSTER3.html]

Details for gene set NB\_KI\_CLUSTER3[GSEA]

|  || Dataset | state33071 |
| Phenotype | NoPhenotypeAvailable |
| Upregulated in class | na\_pos |
| GeneSet | NB\_KI\_CLUSTER3 |
| Enrichment Score (ES) | 0.56352276 |
| Normalized Enrichment Score (NES) | 4.129887 |
| Nominal p-value | 0.0 |
| FDR q-value | 0.0 |
| FWER p-Value | 0.0 |
Table: GSEA Results Summary

  

Fig 1: Enrichment plot: NB\_KI\_CLUSTER3      
 Profile of the Running ES Score & Positions of GeneSet Members on the Rank Ordered List

  

| PROBE | GENE SYMBOL | GENE\_TITLE | RANK IN GENE LIST | RANK METRIC SCORE | RUNNING ES | CORE ENRICHMENT || 1 | EIF5A |  |  | 1 | 0.691 | 0.0530 | Yes |
| 2 | SNRPC |  |  | 46 | 0.466 | 0.0777 | Yes |
| 3 | TXN |  |  | 47 | 0.465 | 0.1136 | Yes |
| 4 | CYCS |  |  | 59 | 0.453 | 0.1458 | Yes |
| 5 | RPL37A |  |  | 64 | 0.448 | 0.1793 | Yes |
| 6 | MRPL33 |  |  | 87 | 0.423 | 0.2062 | Yes |
| 7 | NDUFA4 |  |  | 140 | 0.382 | 0.2224 | Yes |
| 8 | ACTG1 |  |  | 153 | 0.371 | 0.2480 | Yes |
| 9 | MRPL17 |  |  | 159 | 0.368 | 0.2751 | Yes |
| 10 | SET |  |  | 163 | 0.366 | 0.3025 | Yes |
| 11 | CACYBP |  |  | 165 | 0.364 | 0.3304 | Yes |
| 12 | YBX1 |  |  | 167 | 0.364 | 0.3582 | Yes |
| 13 | PPP1R14B |  |  | 174 | 0.359 | 0.3843 | Yes |
| 14 | NDUFAF8 |  |  | 175 | 0.358 | 0.4120 | Yes |
| 15 | METAP2 |  |  | 236 | 0.318 | 0.4211 | Yes |
| 16 | ENO1 |  |  | 241 | 0.314 | 0.4443 | Yes |
| 17 | SRP14 |  |  | 247 | 0.311 | 0.4671 | Yes |
| 18 | COX5B |  |  | 280 | 0.297 | 0.4818 | Yes |
| 19 | ANAPC11 |  |  | 286 | 0.295 | 0.5033 | Yes |
| 20 | UBB |  |  | 294 | 0.293 | 0.5241 | Yes |
| 21 | HMGN1 |  |  | 300 | 0.290 | 0.5452 | Yes |
| 22 | EIF4EBP1 |  |  | 316 | 0.287 | 0.5635 | Yes |
| 23 | CBWD2 |  |  | 762 | -0.267 | 0.4699 | No |
| 24 | MMS22L |  |  | 1494 | -0.306 | 0.3059 | No |
| 25 | INPP5F |  |  | 1558 | -0.310 | 0.3137 | No |
| 26 | SEC61A2 |  |  | 2286 | -0.356 | 0.1545 | No |
| 27 | FADS1 |  |  | 2588 | -0.381 | 0.1066 | No |
| 28 | FANCL |  |  | 2644 | -0.385 | 0.1223 | No |
| 29 | ZGRF1 |  |  | 2897 | -0.408 | 0.0890 | No |
| 30 | PRR3 |  |  | 3048 | -0.425 | 0.0833 | No |
| 31 | ZNF620 |  |  | 3064 | -0.427 | 0.1125 | No |
| 32 | FSD1L |  |  | 3309 | -0.462 | 0.0855 | No |
| 33 | POLE |  |  | 3335 | -0.468 | 0.1152 | No |
| 34 | NAPB |  |  | 3389 | -0.479 | 0.1386 | No |
Table: GSEA details [plain text format]

  

Fig 2: NB\_KI\_CLUSTER3: Random ES distribution      
 Gene set null distribution of ES for **NB\_KI\_CLUSTER3**
